# Serological Evidence of Brucellosis in Goats in Kaduna North Senatorial District of Kaduna State, Nigeria

**DOI:** 10.1155/2013/963673

**Published:** 2013-05-16

**Authors:** B. Y. Kaltungo, S. N. A. Saidu, A. K. B. Sackey, H. M. Kazeem

**Affiliations:** ^1^Veterinary Teaching Hospital, Ahmadu Bello University, Zaria, Nigeria; ^2^Department of Veterinary Medicine, Ahmadu Bello University, Zaria, Nigeria; ^3^Department of Veterinary Microbiology, Ahmadu Bello University, Zaria, Nigeria

## Abstract

A cross-sectional study was carried out to determine the current status of *Brucella* antibodies in goats in Kaduna North Senatorial District of Kaduna State, Nigeria. A total of 442 serum samples (31 bucks and 411 does) were screened using Rose Bengal plate test (RBPT), serum agglutination test with ethylene diaminetetraacetic acid (SAT-EDTA), and lateral flow assay (LFA). *Results.* The prevalence of *Brucella* antibodies was found to be 25.8%, 11.1%, and 2.5% using RBPT, SAT-EDTA, and LFA, respectively. The prevalence in bucks was 32.3%, 3.2%, and 0.0% and 17.5%, 12.4%, and 3.9% in does using RBPT, SAT-EDTA, and LFA, respectively. The prevalence rates for goats less than one year of age using the tests were 1.5%, 0.0%, and 0.0%. While for those within the age bracket of one to three years, the rates were 19.4%, 10.5%, and 3.5%, respectively. The corresponding values for goats above 3 years of age were 34.2%, 15.2%, and 1.8%, respectively. The prevalence of brucellosis in goats in the study area is high which poses a threat to the development of the livestock industry and is of important zoonotic implications in Nigeria.

## 1. Introduction

Brucellosis is a contagious bacterial infection primarily of livestock [1]. The incidence of the disease in humans is thus closely tied to the prevalence of infection in sheep, goats, and cattle, and to practices that allow exposure of humans to potentially infected animals or their products. Human-to-human transmission of the disease is rare, but the possibility of human-to-human transmission of the organism through bone marrow transplantation, blood transfusion, transmammary route, and sexual intercourse has also been documented [2, 3]. The World Health Organization (WHO) considers brucellosis to be a neglected zoonosis because, despite its widespread distribution and effects on multiple species, it is not prioritized by national and international health systems [4]. The species of *Brucella* which infect livestock and their primary hosts are *B. melitensis* (sheep and goats), *B. abortus* (cattle), *B. suis* (pigs), and *B. ovis* (sheep) [5, 6]. Brucellosis is well known for its effects on the decrease in productivity of infected livestock by causing abortions, reducing fertility, and decreasing milk yield, resulting in substantial economic losses [7, 8]. 

Diverse serological prevalence ranging between 0.20% and 79.70%, has been reported in various parts of Nigeria [9]. The infection has further been reported in various animal species in Nigeria [10–16]. This indicates the importance of the disease in Nigeria. 

The socioeconomic and cultural relationship between these animals and man, especially children and women, and the fear of spread of brucellosis among these animals and people in the study area, should these animals be harboring the disease cannot be overemphasized. Goats are a major source of animal-based protein, especially in the rural Nigeria. Furthermore, goat's milk and cheese are fast gaining acceptance worldwide because of some of its advantages over cow's milk.

Unconfirmed cases of abortion and stillbirth among others in small ruminants are continuously being handled by farmers and animal health workers which apparently may have been caused by members of the *Brucella* species. These and many other factors pose a risk of infection to humans and they call for attention. It is, therefore, imperative to evaluate the status of brucellosis in those species of animals with a view to advising the government and stakeholders of small ruminant production on the possible risks posed by the disease to health.

There is paucity of information on the current status of the disease in goats in Kaduna North Senatorial District of Kaduna State, Nigeria. This study, therefore, was aimed at determining the current status of brucellosis in goat in four Local Government Areas of Kaduna North Senatorial District of Kaduna State, Nigeria.

## 2. Materials and Methods

### 2.1. Study Area

**Figure 1:**
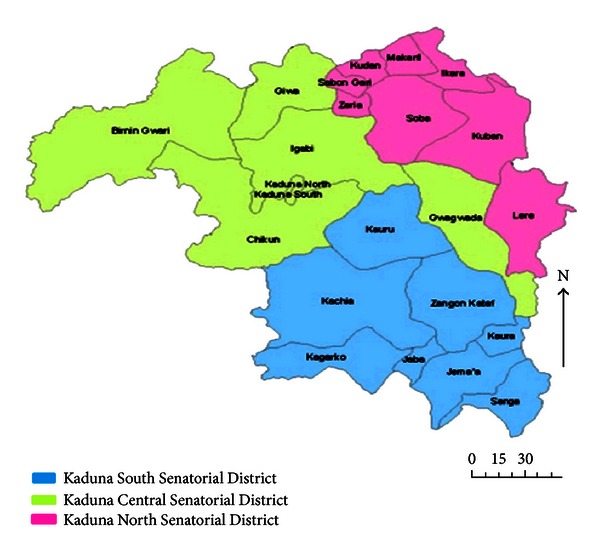
Illustrated map of Kaduna State showing the Senatorial Districts. Source: http://www.ncocusa.com/constituencies_kaduna.html.

The study was conducted in the Kaduna North Senatorial District of Kaduna State is Nigeria (Figure 1). Kaduna State, located in the Northwest Geopolitical Zone of Nigeria. It lies between latitudes 6° and 11° North and longitude 7° and 44° East and is 608 meters above sea level. It has distinct wet and dry seasons and is within the Northern Guinea Savannah zone and part of the Sahel Savannah zone of Nigeria. The state shares geographical boundaries with Katsina and Zamfara States to the North, Plateau and Bauchi States to the East, Nasarawa State and the Federal Capital Territory to the South, Niger State to the West, and Kano State to the Northeast. Kaduna State occupies about 48,473.25 sq.km, with a human population of over 6,066,562 people according to the census figures of 2006 [17].

Four out of the seven LGAs in Kaduna North Senatorial District of Kaduna State were selected using simple random sampling without replacement. They include, Ikara, Makarfi, Sabon Gari, and Soba Local Government Areas (LGAs). The location of the flock, animal breed, age, and sex of each animal sampled were recorded. A total of 442 goats were sampled for the purpose of this study, out of which 31 were bucks and 411 were does.

### 2.2. Study Animals

Pastoral and village level goats were used in this study. The method of flock selection was by random selection and farmers' consent. There was no record of vaccination against brucellosis in any animal species in the area for over twenty years.

### 2.3. Study Design, Collection, and Handling of Blood Samples

The study was carried out between April and May, 2012. Approximately 5 mL of blood was obtained via a jugular venipuncture of apparently healthy goats of all ages, using a 10 mL syringe with 21G needle. The blood samples were then transferred into a well-labeled 10 mL plain blood-collecting tubes and placed in a slanting position under shade to allow it to clot. The samples were then transported to the laboratory in leak proof ice-packed containers, where they were further centrifuged at 1000 g for 5 minutes to allow proper separation of serum from the clotted blood. The serum was then decanted into 5 mL plastic tubes which were properly labelled as for those of the corresponding tubes, after which they were stored in the freezer at −20°C until used.

### 2.4. Serological Tests

Serum samples were tested for *Brucella spp*. antibodies by Rose Bengal plate test (RBPT) as described by [18], serum agglutination test with ethylenediaminetetraacetic acid (SAT-EDTA) as described by [19], and lateral flow assay (LFA) according to the manufacturer's instructions. The antigens for the SAT-EDTA and RBPT were obtained from Onderstepoort Biological Products Ltd., South Africa, while the test kit for the LFA was obtained from Bionote INC., Seogu-dong, Hwaseongi-si, Gyeonggi-do, Republic of Korea.

### 2.5. Rose Bengal Plate Test

Briefly, 30 *μ*L of antigen was placed on a white ceramic tile, and the same volume of 30 *μ*L test serum was placed beside the antigen. The two were mixed thoroughly using sterile applicator stick and rocked gently for 4 minutes and observed for agglutination. The formation of distinct pink granules (agglutination) was recorded as positive, while the absence of agglutination was recorded as negative.

### 2.6. Serum Agglutination Test with Ethylenediaminetetraacetic Acid

Phenol saline with EDTA buffer solution, containing 5 g phenol crystals, 8.5 g sodium chloride, and 1.8612 g disodium EDTA and dissolved in 100 mL of warm distill water was prepared. A 1 : 10 dilution of the concentrated SAT antigen with the prepared buffer with a pinch of 0.02% Safranin O (to provide contrast to the agglutination reaction) was made for each day's work. A 96-well rectangular microtitre plate was set up on the work table. Labeled serum vials were placed on the work table according to positions of the wells, already labeled A–H and a corresponding vertical numbering of the wells. A representative entry of the sample details was made in the laboratory record book. Positive and negative were assigned to row “A,” while rows B–H were designated to the test sera. Using automatic micropipette, 40 *μ*L of the buffer solution was measured out into the first well and 25 *μ*L into each of the remaining microtitre wells. This was followed by the addition of 10 *μ*L of test serum into the first microtitre well using a fresh disposable pipette plastic tip for each test, which was later discarded. A twofold serial dilution was done by transferring 25 *μ*L aliquot from the first well up to the fifth well. 25 *μ*L of the aliquot was discarded after the last well. Contents of the working dilution of the SAT antigen were mixed gently and 25 *μ*L added to each well. Finally, the contents in the microtitre plate were mixed by gently tapping the edges of the plate for 20 seconds. The microtitre plates were covered to prevent evaporation of the contents in the wells and incubated for 20 hours at 37°C in an incubator.

### 2.7. Lateral Flow Assay

 20 *μ*L of thawed serum was placed into the sample hole of the test device, followed by the addition of 4 drops of the provided diluent. Test results were read after 20 minutes by visual inspection for staining of the test and control lines. Tests were scored negative when staining was observed only on the control line, and scored positive when staining was observed on both the test line and control lines.

### 2.8. Statistical Analysis

Data obtained were subjected to statistical analysis using Chi square (chi^2^) test [20]. Values of *P* < 0.05 were considered significant.

## 3. Results

Among the 442 goats sampled for the purpose of this study 114 (25.8%), 49 (11.1%), and 11 (2.5%) were positive for RBPT, SAT-EDTA, and LFA, respectively (Table 1).

Goats sampled from Sabon Gari LGA had the highest RBPT positive results of 16 (28.6%), while goats in Soba LGA had the lowest seroprevalence of 21 (19.1%). Furthermore, the goats in Soba LGA had the highest seroprevalence of 22 (20.0%) when measured with SAT-EDTA, while those in Sabon Gari LGA had the lowest value of 2 (3.6%). With respect to LFA, the highest seroprevalence of 4 (4.8%) was recorded in Ikara LGA, while Sabon Gari LGA had no positive cases. 

Statistical analysis indicated that there was statistical significance in the detection of *Brucella* antibodies using the three serological tests, *P* value < 0.0001, *χ* ^2^ = 107.7, and df = 2.

### 3.1. Seroprevalence Rates per Sex

 The number of goats sampled by sex and their seroprevalence rates for brucellosis using RBPT, SAT-EDTA, and LFA are presented in Table 2. Thirty-one male and 411 female goats were sampled. Out of the 31 male goats tested, seroprevalence of 10 (32.3%), 1 (3.2%), and 0 (0.0%) was recorded when tested with RBPT, SAT-EDTA, and LFA, respectively (Table 2). The highest prevalence of 4 (44.4%) was recorded in Sabon Gari LGAs; none was positive in Soba LGA using RBPT. Similarly, when measured with SAT-EDTA, prevalence of 1 (9.1%) was recorded in Makarfi LGA, while all the other LGAs sampled had no positive cases for *Brucella* antibodies. Similarly, for the LFA, all the LGAs sampled recorded zero seroprevalence for* Brucella* antibodies. 

Four hundred and eleven females were tested, and seroprevalence of 72 (17.5%), 51 (12.4%), and 16 (4.0%) was recorded based on RBPT, SAT-EDTA, and LFA, respectively. The highest seroprevalence of 18 (23.1%) and a corresponding lowest value of 9 (19.2%) were recorded in Ikara and Sabon Gari LGAs, respectively, using RBPT. With respect to SAT-EDTA, the highest seroprevalence of 23 (22.1%) was recorded in Soba LGA, and the lowest value of 2 (4.3%) was recorded in Sabon Gari LGA. Similarly, using the LFA, the highest seroprevalence of 3 (6.4%) was recorded in Sabon Gari LGA and the lowest value of 3 (1.7%) was recorded in Makarfi LGA.

There was no statistical significant difference in seroprevalence rates of *Brucella* antibodies between the male and female animals, *P* = 0.6168.

### 3.2. Seroprevalence Rate per Age

A total of 20 goats of less than one year of age were tested. In this category, only animals from Sabon Gari LGA recorded a seroprevalence of 3 (60%) when tested with RBPT; all the LGAs recorded zero seroprevalence. Zero seroprevalence was also recorded for all the LGAs using SAT-EDTA and LFA, respectively. 

Out of the 258 goats tested within the age bracket of 1–3 years, prevalence of 50 (19.4%), 27 (10.5%), and 9 (3.5%) was recorded based on RBPT, SAT-EDTA, and LFA, respectively. Sabo Gari LGA recorded the highest seroprevalence of 8 (22.2%) in terms of RBPT, while Ikara LGA recorded the lowest value of 6 (13.6%) (Table 3). Similarly, the seropositivity, when tested with SAT-EDTA, was the highest in goats from Soba LGA with a seroprevalence of 15 (18.1%), and lowest value of 1 (2.8%) was recorded from goats in Sabon Gari LGA. Furthermore, Soba LGAs recorded the highest seroprevalence rate in terms of the LFA with the value of 5 (6.0), while zero prevalence was recorded from goats in Sabon Gari LGA.

Similarly, 164 goats above the age of three years were tested and seroprevalence rates of 56 (34.2%), 25 (15.2%), and 3 (1.8%) were recorded, based on RBPT, SAT-EDTA, and LFA, respectively. Soba LGA recorded the highest seroprevalence of 7 (50.0%) in terms of RBPT, while Sabon Gari LGA recorded the lowest value of 2 (13.3%) (Table 3). Similarly, the seropositivity with regards to SAT-EDTA was the highest in Soba LGA with the seroprevalence of 8 (57.1%), and the lowest value of 1 (7.0%) was recorded in goats from Sabon Gari LGA. Furthermore, Ikara LGA recorded the highest seroprevalence of 1 (2.7%) when tested with LFA, while the zero seroprevalence was obtained in Sabon Gari and Soba LGAs, respectively.

Statistical analysis indicated that there was no significant difference in prevalence rates of brucellosis between goats of all age groups tested (*P* = 0.4427, *χ* ^2^ = 1.630, and df = 2).

## 4. Discussion

The present study has established the serological evidence of brucellosis in goats in Kaduna North Senatorial District of Kaduna State. The overall prevalence of 2.5% based on the confirmatory test (LFA) in this work is comparable to that reported by Brisibe et al. [21], where a prevalence of 2.8% was obtained in Northern Nigeria. However, a higher prevalence of 9.0%, 5.88%, and 4.75% was reported in goats by Falade and Shonekan [13], Ogundipe et al. [22], and Shehu et al. [23], respectively. Similarly, an alarming prevalence of 45.75% was reported by Ojo et al. [24] in a goat flock in Abeokuta in western Nigeria. Despite the wide distribution of the sero-prevalence throughout the LGAs sampled, there are some differences between the Local Government Areas which may be attributed to the migratory habit of the Fulani pastoralists. This observation may account for infection in small ruminants since they are allowed to mix freely with cattles, and it was reported that there is an increase in seroprevalence of brucellosis among the Fulani pastoralists' cattles [25]. The difference in the seroprevalence obtained by different workers may be due to sensitivities and specificities of the different diagnostic methods used among the researchers. 

The present study also shows that more animals were seropositive with RBPT and SAT-EDTA as compared with the LFA. The high seroprevalence rate from the RBPT may be attributed to the relatively low specificity and very high sensitivity of the test. Apparently, it could also as a result of reaction to other SLPS *Brucella* species, especially *B. abortus* since. This is because goats in the study area are herded or kept together with cattle. It could also be due to other Gram-negative bacteria like *Vibrio cholerae* O1, *Escherichia coli *O: 157, some strains of *Escherichia hermannii *and* Stenotrophomonas maltophilia, Salmonella* group N (O: 30), and *Yersinia enterocolitica* O: 9 which has LPS O-chains similar to those of brucellae. These organisms have agglutinins capable of reacting with *Brucella* antigens, thus giving false positive reactions. Despite these limitations, the RBPT may be used as a screening test to ascertain exposure of animals to infection due to *Brucella *species. 

The seroprevalence rate was lower with the SAT-EDTA, and this finding may be attributed to the increase in specificity of this test. The result agrees with the finding of Bertu et al. [26] where prevalence of 9.3% and 5.2% with RBPT and SAT was obtained, respectively. The SAT-EDTA was particularly more specific because of the addition of ethylenediaminetetraacetic acid (EDTA) which increases the specificity of the test by eliminating nonspecific agglutination reactions, apparently by preventing binding between *Brucella* cell surface components and the Fc portion of IgM [27].

The least seroprevalence rate recorded with the LFA was indicative of its very high specificity since it only detects antibodies due to *B. melitensis.* Due to the high sensitivity, specificity, and simplicity of the test and especially that the test does not involve any expertise nor refrigeration, it is recommended that this assay should be used for serological survey of Brucellosis I in Nigeria, particularly in the rural areas.

From the study, female animals were more seropositive than their male counterparts. This could be due to the fact that more females were available for sampling as they are kept in the flock for a longer period for the purpose of breeding. This is in agreement with the work of Mohammed et al. [28] where it was reported that female animals were more seropositive than males. Generally, goat farmers keep fewer males because of their aggressive nature and mainly for breeding the females. Furthermore, the higher prevalence may be because female goats are more susceptible than males as reported by Keppie et al. [29]. 

The animals within the age of 1–3 years of both sexes had the highest seroprevalence rate. This finding is in agreement with the results obtained by Mohammed et al. [28], Aulakh et al. [30], and Abubakar et al. [31], who reported that the incidence is higher in sexually mature animals. Furthermore, in this study, more animals within this age bracket were sampled. The animals within this age range are actively involved in breeding. Therefore, the presence of brucellosis in them may result in serious economic loss in terms of reproductive wastages like abortion, still-birth, infertility, sterility, and reduced milk production. It also means that they are capable of spreading the infection since they mix among themselves from different flocks. It is also important to note that animals in this age bracket are more often sold out for slaughter and may pose serious risk to humans as a source of infection.

Animals of less than 1 year of age that were seropositive may have been exposed through suckling of their infected dams. They may also have been infected through contaminated pasture or water at grazing and watering points. Infection of goats of this age is an evidence of their potential to develop the disease and consequently spread it to others.

As for goats that were over 3 years old, the high prevalence recorded in them was because the animals have acquired the infection much earlier in life.

## 5. Conclusion

The present study has shown that brucellosis exists in goats in the study area. It has also indicated that RBPT is an important screening test for brucellosis and that the LFA is a reliable test for identification of *B. melitensis.* Furthermore, the female goats were found to be more affected than their male counterparts, and goats within the age of 1–3 years are more affected. In view of the importance of brucellosis to the livestock industry and its zoonotic importance, government should institute stringent control measures and possible eradication strategies of the disease.

## Figures and Tables

**Table 1:** Distribution of brucellosis seroprevalence in goats in four Local Government Areas in Kaduna North Senatorial District of Kaduna State, Nigeria.

LGA	Number of samples	RBPT (%)	Positive SAT-EDTA (%)	LFA (%)
Ikara	83	22 (26.5)	12 (14.5)	4 (4.8)
Makarfi	193	55 (28.5)	13 (6.7)	3 (1.6)
Sabon Gari	56	16 (28.6)	2 (3.6)	0 (0.0)
Soba	110	21 (19.1)	22 (20.0)	4 (3.6)

Total	442	114 (25.8)	49 (11.1)	11 (2.5)

RBPT: Rose Bengal plate test.

SAT-EDTA: serum agglutination test with ethylene diaminotetra acetic acid.

LFA: lateral flow assay.

LGA: Local Government Area.

**Table 2:** Prevalence of Brucellosis in male and female goats in Kaduna North Senatorial District of Kaduna State, Nigeria.

LGA	Sex							
	Tested	Male Positive (%)			Tested	Female Positive (%)		
		RBPT	SAT-EDTA	LFA		RBPT	SAT-EDTA	LFA
Ikara	5	2 (40.0)	0 (0.0)	0 (0.0)	78	18 (23.1)	13 (17.0)	4 (5.1)
Makarfi	11	4 (36.4)	1 (9.1)	0 (0.0)	182	51 (20.0)	13 (7.1)	3 (1.7)
Sabon Gari	9	4 (44.4)	0 (0.0)	0 (0.0)	47	9 (19.2)	2 (4.3)	3 (6.4)
Soba	6	0 (0.0)	0 (0.0)	0 (0.0)	104	21 (20.2)	23 (22.1)	5 (4.8)

Total	31	10 (32.3)	1 (3.2)	0 (0.0)	411	72 (17.5)	51 (12.4)	16 (4.0)

RBPT: Rose Bengal plate test.

SAT-EDTA: serum agglutination test with ethylene diaminotetra acetic acid.

LFA: lateral flow assay.

LGA: Local Government Area.

**Table 3:** Prevalence of Brucellosis in goats per different age groups in Northern part of Kaduna State.

LGA	Age range (yrs)											
	<1				1–3				>3			
	Tested	Positive (%)			Tested	Positive (%)			Tested	Positive (%)		
		RBPT	SAT-EDTA	LFA		RBPT	SAT-EDTA	LFA		RBPT	SAT-EDTA	LFA
Ikara	2	0 (0.0)	0 (0.0)	0 (0.0)	44	6 (13.6)	5 (11.4)	3 (7.0)	37	14 (37.8)	8 (22.0)	1 (2.7)
Makarfi	0	0 (0.0)	0 (0.0)	0 (0.0)	95	22 (21.2)	6 (6.3)	1 (1.1)	98	33 (34.0)	8 (8.2)	2 (2.0)
Sabon Gari	5	3 (60.0)	0 (0.0)	0 (0.0)	36	8 (22.2)	1 (2.8)	0 (0.0)	15	2 (13.3)	1 (7.0)	0 (0.0)
Soba	13	0 (0.0)	0 (0.0)	0 (0.0)	83	14 (17.0)	15 (18.1)	5 (6.0)	14	7 (50.0)	8 (57.1)	0 (0.0)

Total	20	3 (15.0)	0 (0.0)	0 (0.0)	258	50 (19.4)	27 (10.5)	9 (3.5)	164	56 (34.2)	25 (15.2)	3 (1.8)

RBPT: Rose Bengal plate test.

SAT-EDTA: serum agglutination test with ethylene diaminotetra acetic acid.

LFA: lateral flow assay.

LGA: Local Government Area.
